# Mouth-nose masks impair the visual field of healthy eyes

**DOI:** 10.1371/journal.pone.0251201

**Published:** 2021-05-13

**Authors:** Annika Weber, Bettina Hohberger, Antonio Bergua

**Affiliations:** Department of Ophthalmology, University of Erlangen, Friedrich-Alexander-Universität Erlangen-Nürnberg (FAU), Erlangen, Germany; The University of Melbourne, AUSTRALIA

## Abstract

**Background:**

Mouth-nose masks have been requested to prevent the transmission of severe acute respiratory syndrome coronavirus-2 (SARS-CoV-2). The aim of the present study was to investigate, if wearing a mouth-nose mask impairs the visual field function in normals.

**Methods:**

Thirty eyes of 30 subjects were recruited for the present study. White-on-white perimetry (OCTOPUS 900; 90°) was done and sensitivity was analysed in 14 defined test points (P1-P14, inferior visual field) under 3 different test conditions while the subjects were wearing a mouth-nose mask: (I) 1.5 cm under the lower eyelid, nose clip not used (position_1.5cm_no_clip_); (II) 1.5 cm under the lower eyelid, nose clip correctly positioned (position_1.5cm_with_clip_); (III) 0.5 cm under the lower eyelid, nose clip correctly positioned (position_0.5cm_with_clip_). All data were compared to sensitivity without wearing a mouth-nose mask (reference). Mean Δ was calculated, being the difference between the results of each test condition and reference, respectively.

**Results:**

Sensitivity was significantly different between position_1.5cm_no_clip_ and reference at 10 test points (p<0.05). Sensitivity at test point P7 was significantly different between position_1.5cm_with_clip_ and position_0.5cm_with_clip_ compared to reference (p<0.001), respectively. Mean Δ increased while wearing a mask at P7: position_1.5cm_with_clip_ (-8.3 dB ± 7.3 dB) < position_0.5cm_with_clip_ (-11.3 dB ± 9.5 dB) < position_1.5cm_no_clip_ (-20.1 dB ± 7.6 dB).

**Conclusion:**

Visual field function was observed to be significantly impaired in the inferior-nasal sector while persons were wearing a mouth-nose mask, especially when the nose clip was not correctly used.

## Introduction

Visual field can be impaired due to diverse diseases mostly by ocular (e.g. glaucoma [1, 2], retinitis pigmentosa [3], diabetic retinopathy [4]) and neurological disorders (e.g. stroke [5], cancer [6]). A perimetric defect can affect central or peripheral visual field or even both. Visual field defects can be measured by using a static or kinetic perimeter. Static perimetry is the most commonly used type of perimetry, using fixed test points. The stimuli are presented in different luminance in order to find each sensitivity for each defined point. Contrary, the stimulus is moving from the non-seen-region into the seen-region along a vector in the kinetic perimetry. Static perimetry is more suitable for detecting small changes of sensitivity, especially in the central part of the visual field, than kinetic ones. Therefore, it is used more commonly for diagnosis of diseases with a more central affection (e.g. glaucoma) [7]. It is important to analyse the functional impact of a disorder without any side-effects. This enables an exact initial diagnosis and unbiased follow-up. As perimetry is a psychophysical method, it is known that the results can be affected by e.g. concentration [8] mental disability or even wrong refraction [9]. In addition, extraocular factors (e.g. malposition of eyelids [10]) can affect visual field function, consequently interfering with the functional deficit of ocular diseases.

Coronavirus Disease-2019 (COVID-19) has reached pandemic character in 2020. Severe acute respiratory syndrome coronavirus-2 (SARS-CoV-2) was assigned as causing agent, transmitted from human-to-human mostly via the respiratory system [11, 12]. In order to prevent or at least to reduce infection with SARS-CoV-2 via respiratory tract, the World Health Organization recommends to keep physical distance of at least one metre and to wear a mask covering mouth and nose [13]. The protective efficiency of the masks [14] is even higher when both, the not infected person and the virus spreader, are wearing it [15]. People in many countries have therefore been bound by law to wear a mouth-nose mask in situations when it is not possible to keep distance. There has been no defined standard except that the mask had to cover mouth and nose. Therefore, people have been wearing many different kinds of masks, sometimes home-made or ill-fitting. Two case reports have already reported about visual field artefacts from using a mouth-nose-mask in clinical visual field testing by fogging of the trial lens or incorrect wearing of the mask [16, 17]. Considering different sizes and shapes of the mouth-nose-masks, there might be different effects of visual field restriction. To the best of our knowledge there is no study available in literature investigating a potential impact of mouth-nose-masks on visual field function in healthy eyes considering different positions of the masks. Thus, it was the aim of the present study to investigate, if wearing a mouth-nose-mask in different positions can affect visual field function in normals.

## Material and methods

### Participants

Thirty eyes of 30 participants (19 male; 11 female) were included in the present study. The average age was 25.4 years with a range of 20–37 years. All participants received a complete standardized ophthalmologic examination including slit-lamp microscopy, funduscopy and non-contact-tonometry. The presence of any eye disease or previous ophthalmologic surgery (except for laser treatment) was an exclusion criterion. Best corrected visual acuity was ≥0.8 (decimal) or 20/25 (Snellen notation). Intraocular pressure was within the normal range (≤21 mmHg). All participants had to do a test run before participating and the order of the measurements was different between each participant in order to avoid learning effects. The study protocol has been approved by the local ethic committee of the University of Erlangen-Nürnberg and has been performed in accordance with the tenets of the Declaration of Helsinki. Informed written consent has been obtained from all participants.

### Mouth-nose mask

**Fig 1:**
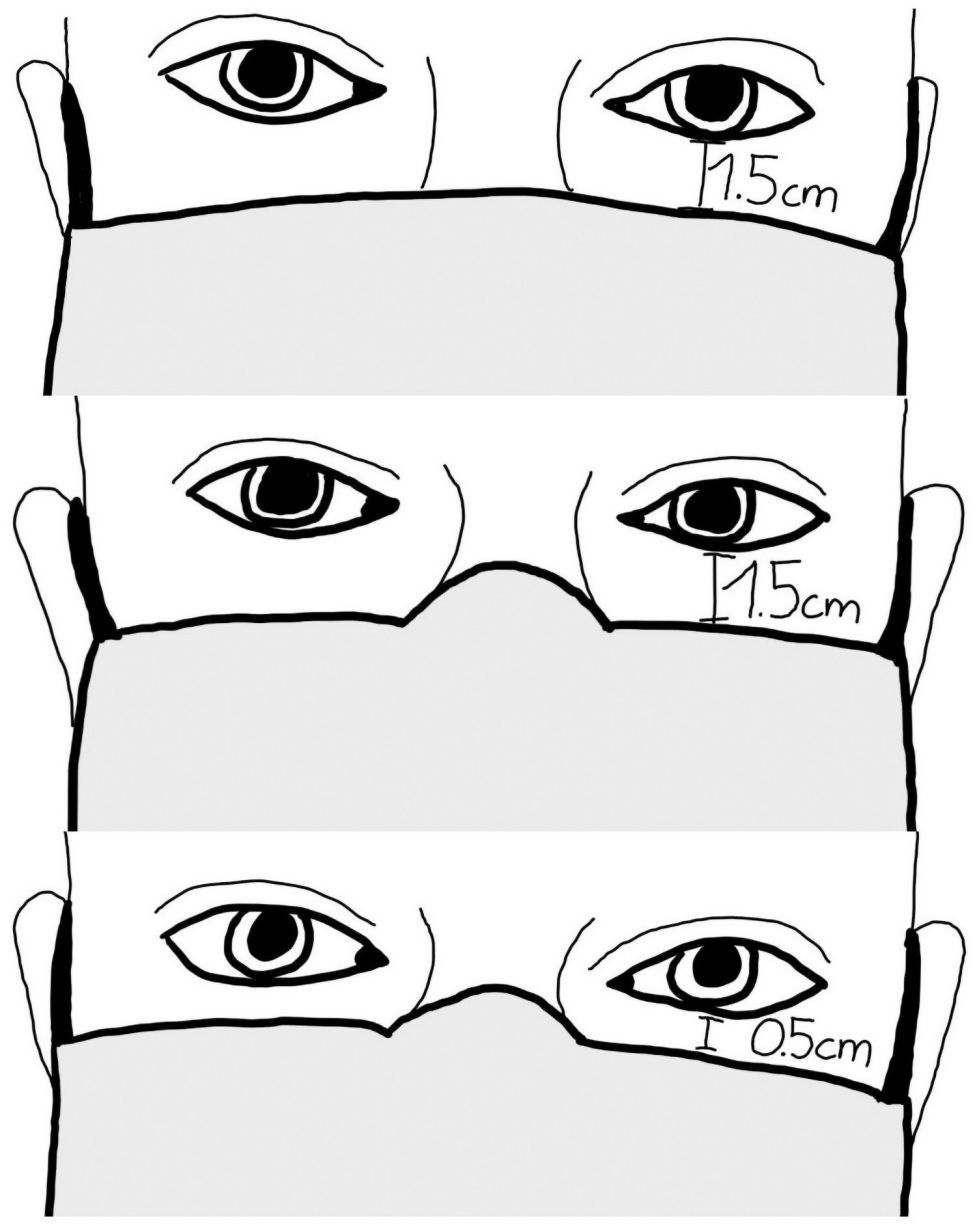
Position of the mouth-nose mask during visual field testing. Position_1.5cm_no_clip_ (top): 1.5 cm below the lower eyelid without using the nose clip; position_1.5cm_with_clip_ (middle): 1.5 cm below the lower eyelid with use the nose clip; position_0.5cm_with_clip_ (bottom): 0.5 cm below the lower eyelid using the nose clip.

A medical mouth-nose mask (Mölnlycke BARRIER Medical Face Mask, Göteborg, Sweden) was used. The mask had head ties for fixation behind the head and a nose clip. Positioning the mask in three different ways (Fig 1) resulted in four different measurements per participant including the reference measurement without wearing a mask. Tape was used to fixate the mask to avoid the mask riding up the participant’s face and to keep the mask in the required place. The first position of the mask was 1.5 cm under the lower eyelid without using the nose clip (position_1.5cm_no_clip_). The second position was 1.5 cm under the lower eyelid while using the nose clip correctly (position_1.5cm_with_clip_). The third position was 0.5 cm under the lower eyelid while using the nose clip correctly (position_0.5cm_with_clip_). The order of the measurements was chosen randomly.

### Perimetry

Visual field function has been tested by white-on-white perimetry (OCTOPUS 900, Haag-Streit, Switzerland; EyeSuite V3.6.1) using the Low-Vision Strategy. This strategy is a quantitative strategy, allowing to detect specific sensitivity for each point with a high accuracy.

**Fig 2:**
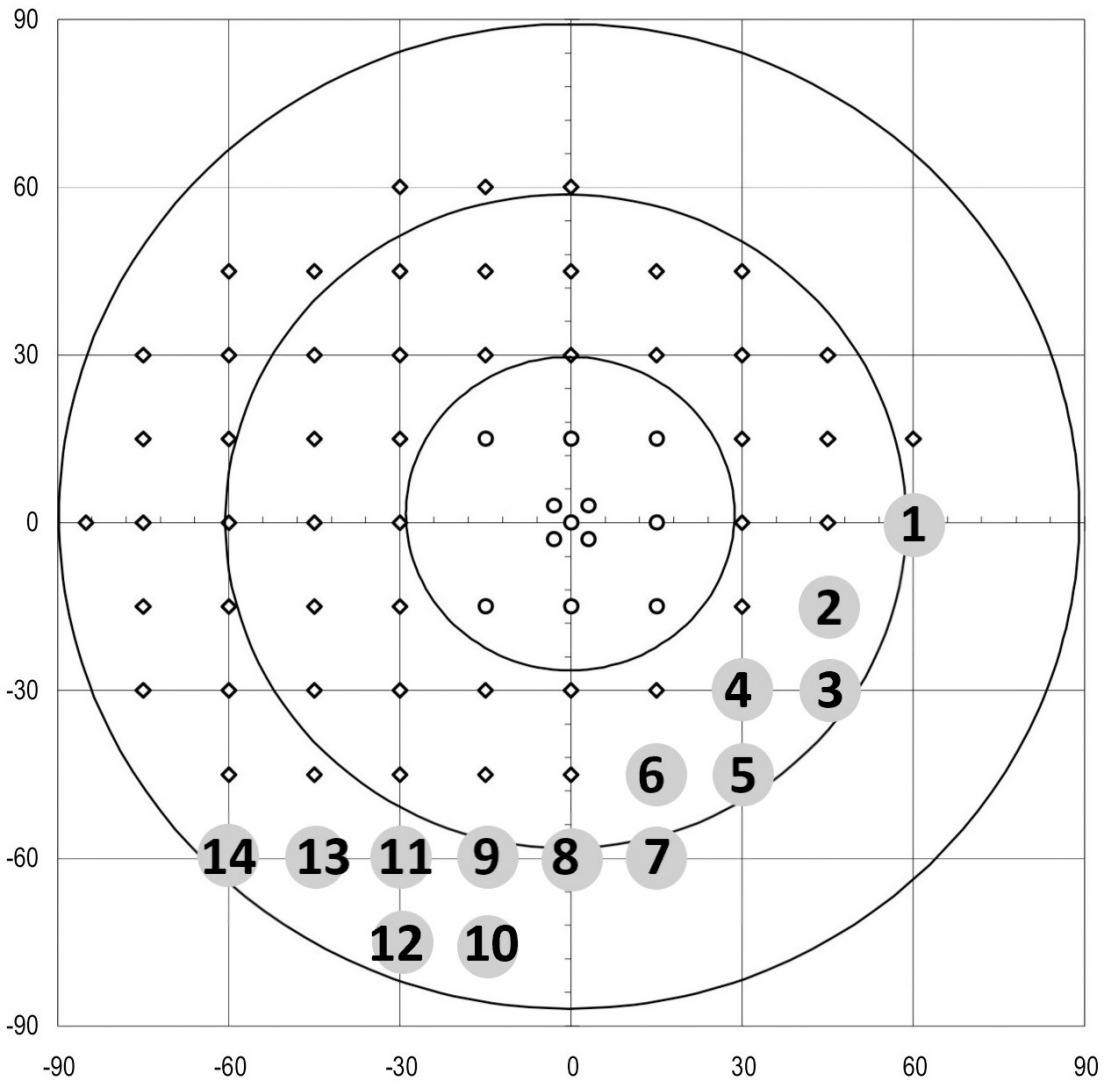
90° perimetry, displaying the 14 defined test-points.

The stimulus is round with an area of 64 mm^2^, being similar to the Goldmann Stimulus size V and presented with a length of 200 ms. The maximum stimulus luminance is 0 dB (= 4000 asb) and the lowest is 40 dB (= 0.4 asb) with a background luminance of 31.4 asb. Using the 90° perimetry Low-Vision strategy, it was not necessary to use a trial lens. As no lens was used, invalid data due to fogging were avoided. The test was done monocularly. The second eye was occluded with an eye patch. Fourteen points in the quantitative visualisation were defined in order to compare potential perimetric restrictions (Fig 2). The cartesian coordinates for the left eye are: P1 (60|0), P2 (45|-15), P3 (45|-30), P4 (30|-30), P5 (30|-45), P6 (15|-45), P7 (15|-60), P8 (0|-60), P9 (-15|-60), P10 (-15|-75), P11 (-30|-60), P12 (-30|-75), P13 (-45|-60), P14 (-60|-60). Each test point was compared between the test run while wearing a mask and without wearing a mask, respectively.

The rate of false positive answers, detecting participants who respond without a stimulus, had to be ≤ 20%. The rate of false negative answers, detecting loss of attention, had to be ≤25%. The reliability factor had to be <18% per measurement and the overall reliability factor (calculated of all four measurements) had to be <10%. The reliability factor was calculated as the sum of false positive and false negative answers.

### Statistical analysis

Statistical analysis was done by Microsoft Excel version 2010 and SPSS 2020. Mean values and standard deviations were calculated. Quantitative analysis showed sensitivity [dB] for each test point: test points P1-P14 were compared between the run without wearing the mask and while wearing the mask in the three different positions, respectively. The differences in sensitivity (Δ) at the 14 test points compared to the reference points were calculated for each participant and each mask position. Furthermore, position_1.5cm_no_clip_ and position_0.5cm_with_clip_ was compared to position_1.5cm_with_clip_ in the same way. A Wilcoxon signed-rank test was done (level of significance, p<0.05). Bonferroni correction was done considering multiple testing.

## Results

**Fig 3:**
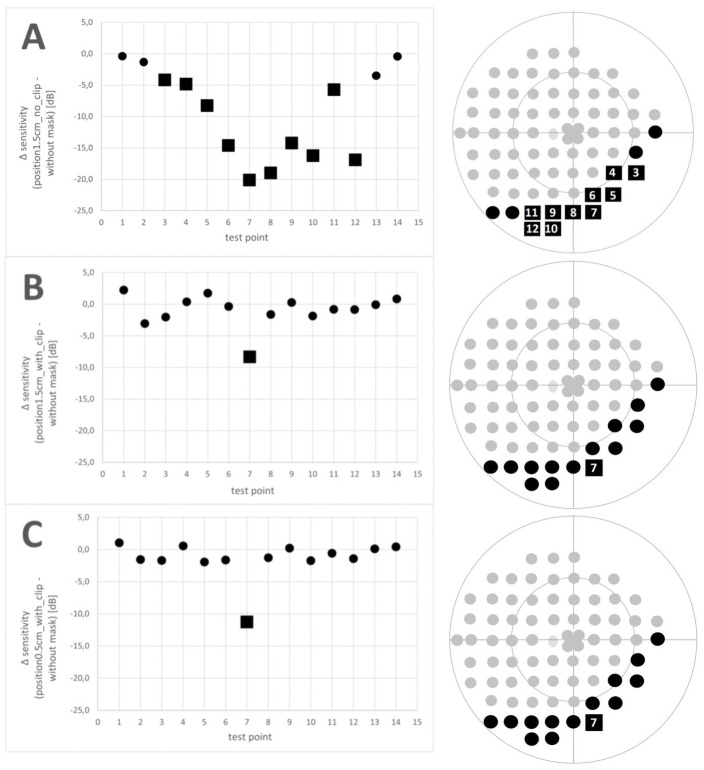
Differences in sensitivity between the different mask positions (A: Position_1.5cm_no_clip_; B: Position_1.5cm_with_clip_; C: Position_0.5cm_with_clip_) and without wearing a mask (Δ): Mean Δ sensitivity; the square represents significant differences (p<0.05), the circle represents no significant difference; significant reductions Δ were observed for P7 (all three positions) and P8 (position_1.5cm_no_clip_).

Mean sensitivity of the overall perimetry was 28.5±2.4 dB without wearing a mask, 26.6±2.4 dB while wearing the mask in position_1.5cm_no_clip_, 28.3±2.4 dB while wearing the mask in position_1.5cm_with_clip_, and 27.8±2.2 dB while wearing the mask in position_0.5cm_with_clip_. Mean sensitivity at each test point can be seen in Table 1. Detailed analysis for each test point yielded that the sensitivity at test point P3 –P12 was significantly different between position_1.5cm_no_clip_ compared to the sensitivity without wearing a mask (Fig 3). In addition, sensitivity at test point P7 differed significantly between wearing a mask in position_1.5cm_with_clip_ (p<0.001) or in position_0.5cm_with_clip_ (p<0.001) and without wearing a mask, respectively. No significant differences in sensitivity were observed at test point P1, P2, P13 and P14 (position_1.5cm_no_clip_) and at test point P1-P6 and P8-P14 (in position_1.5cm_with_clip_ and position_0.5cm_with_clip_), compared to sensitivity without wearing a mask (p>0.05).

**Table 1:** Mean sensitivity.

Test point	Mean sensitivity without mask [dB]	Mean sensitivity position_1.5cm_no_clip_ [dB]	Mean sensitivity position_1.5cm_with_clip_ [dB]	Mean sensitivity position_0.5cm_with_clip_ [dB]
**P1**	18.8	18.4	21.0	19.8
**P2**	28.6	27.2	25.5	27.0
**P3**	18.7	14.5	16.6	17.0
**P4**	29.2	24.4	29.6	29.8
**P5**	21.2	12.9	22.9	19.3
**P6**	30.0	15.4	29.6	28.4
**P7**	27.2	7.0	18.8	15.9
**P8**	28.4	9.4	26.7	27.1
**P9**	28.3	14.1	28.6	28.6
**P10**	23.4	7.2	21.6	21.7
**P11**	29.1	23.3	28.3	28.5
**P12**	23.6	6.7	22.7	22.2
**P13**	27.5	24.0	27.4	27.6
**P14**	13.7	13.3	14.5	14.1

Mean sensitivity in the 14 test points in all four measurements.

Differences in sensitivity between the different mask positions and reference showed a maximum Δ at test point P7 (all three positions) and P8 (position_1.5cm_no_clip_ Fig 3). Especially at test point P7, mean Δ increased while wearing a mask in: position_1.5cm_with_clip_ (mean Δ -8.3±7.3 dB) < position_0.5cm_with_clip_ (mean Δ -11.3±9.5 dB) < position_1.5cm_no_clip_ (mean Δ -20.1±7.6 dB).

**Fig 4:**
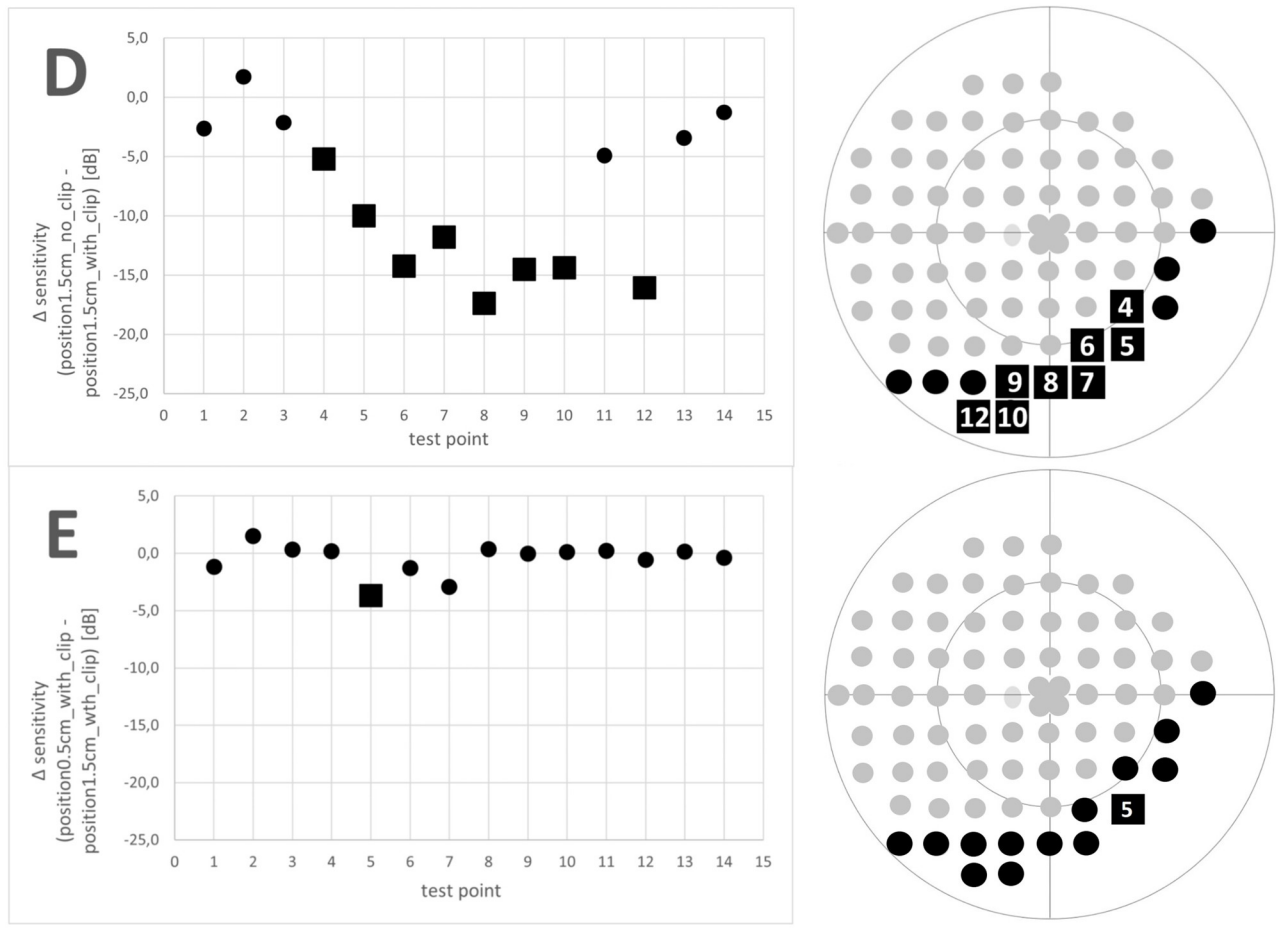
Differences in sensitivity between D: Position_1.5cm_no_clip_ compared to position_1.5cm_with_clip_ and E: Position_0.5cm_with_clip_ compared to position_1.5cm_with_clip_; mean Δ sensitivity; the square represents significant differences (p<0.05); the circle represents no significant difference.

Comparing the sensitivity between position_1.5cm_with_clip_ and position_0.5cm_with_clip_, significant differences (p = 0.015) were observed in test point P5 due to different positions under the lower eyelid (Fig 4). Data of not using the nose clip yielded significant differences in 8 of 14 test points (comparison of position_1.5cm_with_clip_ and position_1.5cm_no_clip_, Fig 4).

## Discussion

COVID-19 pandemic required wearing mouth-nose masks in order to prevent or at least to reduce the risk of an infection with SARS-CoV-2. A huge variety of mouth-nose masks is available. In addition, many people have manufactured and have been wearing their own home-made cotton masks. Most of the home-made cotton masks do not have a nose clip and thus cannot be worn closely to the nose. Wearing a mask in the wrong way (e.g. without using the nose clip at all) or wearing masks that have no nose clip or that cannot be adjusted to the nose might affect the visual field. The data of the present study showed that visual field function was significantly impaired in 10 of 14 test points while wearing a mask 1.5 cm below the lower eyelid without using the nose clip. These test points represent the lower part of the visual field, which is especially important for orientation, walking and driving. Thus, even if the subject was wearing the mask in the right way (i.e. using the head ties for fixation behind the head and the nose clip to fix the mask closely to the face), a significantly impaired visual field was observed in one test point of the present study. The deviation to the reference measurement was even higher when the mask was worn only 0.5 cm under the lower eyelid (position_0.5cm_with_clip_). Wearing the mask 1.5 cm under the lower eyelid (position_1.5cm_with_clip_) impaired visual field as well less, being probably due to the adjusted nose clip. Yet, even when the nose clip was adjusted in the right way and pressed closely to the nose, it still represented an obstruction in the lower nasal visual field.

To the best of our knowledge, the data of the present study are the first ones investigating an impairment of visual field due to mouth-nose-masks in healthy eyes. Only two recent case reports had previously shown visual field artefacts from using a mouth-nose-mask in clinical visual field testing due to fogging of the trial lens [16, 17]. The mask can be taped to the nose [16] or can be tied around the head with a special technique, in which the superior and inferior ties cross before the ear in order to reduce fogging [18]. In one of the case studies the examiners perceived that due to the mask riding up in the face during the examination the patient’s visual field was reduced [17]. Many ocular and neurological diseases impair visual field. Their visual field will be even more restricted, if these patients do wear a mask in the wrong way or wear a mask that cannot be worn closely to the nose. An enhanced visual field loss can result in a higher risk of falling [19–21]. Incidents like falling might increase morbidity and mortality especially in elderly persons [22]. Perimetric loss in the inferior visual field was associated with a poorer functional status. Weaker lower limb strengths and slower timed-up and go performance [23], a slower walking speed [24], and shortened step length [25] were reported. In addition, restriction of visual field is a problem considering traffic [20, 26, 27]. People with visual field defects often use compensatory mechanisms like moving their eyes more frequently [28]. This compensatory mechanism is useful and reduces the number of collisions, but it cannot prevent all collisions. A study with young and healthy participants showed an increasing number of pedestrian collisions after constricting the participants visual field. Using compensatory mechanisms (e.g. eye movements) reduced these pedestrian collisions, yet the number of collisions was still significantly increased compared to without restriction of visual field [29]. Therefore, wearing a mask while driving a car might be a reason for preventable accidents, even if the driver is young and healthy and uses compensatory mechanisms.

The present study is not without limitations. The data could be biased by the not systematic and principled approach of randomisation of the order of measurements (with the various mask positions or selection of study eye). Further on, only one type of mouth-nose mask was used during the tests. As several types of mouth-nose masks are available (e.g. home-made, FFP2, FFP3) it would be of interest, if other types of masks might impair visual field in the same way or even more. In addition, testing mask-induced visual field impairment would be of interest in patients with pre-existing perimetric loss.

## Conclusion

The data of the present study showed that it is important to wear the mouth-nose mask correctly in order to avoid a perimetric impairment. While manufacturing own home-made masks, it should be kept in mind to use a pattern that will not be an obstruction in the field of view and include a nose clip. However, even if the mask is correctly fixed to the head, the mask was observed to be still a factor influencing visual field function. Therefore, it should be considered, if wearing a mask while driving a car is sensible: a plastic shield between driver and passengers can be an option to avoid a mask-induced restricted visual field of the driver and would therefore contribute to safety of driver and passengers.
